# The J-shape association of ethanol intake with total homocysteine concentrations: the ATTICA study

**DOI:** 10.1186/1743-7075-1-9

**Published:** 2004-10-14

**Authors:** Christos Pitsavos, Demosthenes B Panagiotakos, Meropi D Kontogianni, Christina Chrysohoou, Yannis Chloptsios, Antonis Zampelas, Antonia Trichopoulou, Christodoulos Stefanadis

**Affiliations:** 1 First Cardiology Clinic, School of Medicine, University of Athens, Athens, Greece; 2 Department of Dietetics and Nutrition, Harokopio University, Athens, Greece; 3 Department of Hygiene and Epidemiology, School of Medicine, University of Athens, Athens, Greece

**Keywords:** ethanol, homocysteine, inflammation

## Abstract

**Background:**

Epidemiological studies suggest a non-monotonic effect of alcohol consumption on cardiovascular risk, while there is strong evidence concerning the involvement of homocysteine levels on thrombosis. The aim of this work was to evaluate the association between usual ethanol consumption and homocysteine levels, in cardiovascular disease free adults.

**Methods:**

From May 2001 to December 2002 we randomly enrolled 1514 adult men and 1528 women, without any evidence of cardiovascular disease, stratified by age – gender (census 2001), from the greater area of Athens, Greece. Among the variables ascertained we measured the daily ethanol consumption and plasma homocysteine concentrations.

**Results:**

Data analysis revealed a J-shape association between ethanol intake (none, <12 gr, 12 – 24 gr, 25 – 48 gr, >48 gr per day) and total homocysteine levels (mean ± standard deviation) among males (13 ± 3 vs. 11 ± 3 vs. 14 ± 4 vs. 18 ± 5 vs. 19 ± 3 μmol/L, respectively, p < 0.01) and females (10 ± 4 vs. 9 ± 3 vs. 11 ± 3 vs. 15 ± 4 vs. 17 ± 3 μmol/L, respectively, p < 0.01), after controlling for several potential confounders. The lowest homocysteine concentrations were observed with ethanol intake of < 12 gr/day (Bonferroni α* < 0.05). No differences were observed when we stratified our analysis by type of alcoholic beverage consumed.

**Conclusion:**

We observed a J-shape relationship between homocysteine concentrations and the amount of ethanol usually consumed.

## Introduction

Alcoholic beverages are widely consumed throughout the world and it has long been known that heavy alcohol consumption is hazardous to various body organs. In several countries alcohol is considered as one of the leading causes of preventable deaths, after smoking [1]. However, there is now also substantial evidence that the intake of light to moderate amounts of ethanol is associated with reduced morbidity and mortality from several cardiovascular conditions, particularly coronary heart disease (CHD) [2]. The interpretation of these beneficial effects has been extensively discussed and it has been suggested that the effects on cardiovascular disorders might not be due to ethanol per se but to other confounding factors [3]. Low to moderate ethanol consumption has been associated with reduced mortality, primarily due to a reduction in coronary heart disease (CHD). Conversely, heavy drinking increases mortality, mainly due to haemorrhagic stroke and non-cardiovascular diseases [4,5].

Some investigators consider increased homocysteine levels as an independent risk factor of cardiovascular disease, and its involvement in mechanisms of thrombosis has well been documented [6,7]. Moreover, other studies suggest that an elevated plasma total homocysteine concentration increases the risk associated with some of the conventional cardiovascular risk factors [8,9]. However, there are findings that do not confirm or recognize homocysteine importance in actually causing coronary artery disease, while recent studies have considered homocysteine more as a result than a cause of arteriosclerosis, especially due to the confounding effect of various nutrients and other lifestyle-related factors, including alcohol drinking [10-13].

We therefore studied the relation between amount of ethanol consumption and homocysteine levels, in 3042 adults enrolled in the ATTICA Study.

## Subjects and Methods

### Study population

The "ATTICA" study [14] is a health and nutrition survey, which is being carried out in the province of Attica (including 78% urban and 22% rural areas), where Athens is the metropolis. The sampling was random, multistage and it was based on the age – sex distribution of the province of Attica, provided by the National Statistical Service (census of 2001). Also, all people living in institutions were excluded from the sampling, and we enrolled only one participant per household. From May 2001 to December 2002, 4056 inhabitants from the above area, who had no clinical symptoms or signs of cardiovascular or any other atherosclerotic disease (as assessed by the physical examination and reported medical history), nor evidence of chronic viral infections, were randomly selected to enter into the study. None of the participants was under current or chronic use of certain drugs that influence homocysteine levels, like methotrexate, trimethoprin, cholestyramine and cyclosporine. Moreover, subjects did not have cold or flu, acute respiratory infection, dental problems or any type of surgery in the preceding week. Of the 4056 inhabitants, 1518 men (46 ± 13 years old) and 1524 (45 ± 13 years old) women agreed to participate (75% participation rate). Participants were interviewed by trained personnel (cardiologists, general practitioners, dieticians and nurses) who used a standard questionnaire.

The selected sample was population-based and reflecting the underlying population with respect to sex, age and residence. The number of the participants was determined by power analysis and chosen to evaluate greater than 0.5 standardised differences between ethanol groups and homocysteine levels, with statistical power > 0.80 at < 0.05 probability level (P-value).

### Measurements

The questionnaire included demographic characteristics (age, sex, mean annual income and years of school), detailed medical history and lifestyle habits, such as food items consumed, smoking habits and physical activity status.

Dietary intake during the year before enrolment was assessed through a semi-quantitative food frequency questionnaire provided by the EPIC-Greece Study. The questionnaire was administrated in person by specially trained dieticians and has been validated [15]. The daily ethanol intake was assessed in a 7-day food record. All alcoholic beverages consumed, i.e. wine, beer, whisky, traditional alcoholic drinks, like "retsina" or "tsipouro", and other spirits were recorded and daily ethanol intake (in grams) was calculated. For the presentation of our findings we categorized ethanol intake into five groups: (a) no ethanol intake, (b) low (< 12 gr), (c) moderate (12 – 24 gr), (d) high (25 – 48 gr) and (e) very high (>48 gr). Moreover, the frequency of consumption of several food groups was quantified approximately in terms of the number of times per month the food was consumed.

Regarding the rest of the investigated parameters the educational level of the participants (as an index of social status) was measured in years of school. Information about smoking habits was collected using a standardized questionnaire developed for the Study. Current smokers were defined as those who smoked at least one cigarette per day. Former smokers were defined as those who had stopped smoking more than one year previously. The rest of the participants were defined as non smokers. For the multivariate statistical analyses cigarette smoking was quantified in pack-years (cigarette packs per day × years of smoking), adjusted for a nicotine content of 0.8 mg / cigarette. All participants were classified at entry according to their habitual physical activity. Class 1 were sedentary, engaging in little exercise; class 2 were moderately active during a substantial part of the day; and class 3 performed hard physical work much of the time. Classification was based on the responses to questions about the occupation and usual activities, including part-time jobs and notable non-occupational exercise [14]. Body mass index was measured as weight (in kilograms) divided by standing height (in meters squared). Obesity was defined as body mass index > 29.9 Kg / m^2^.

Blood samples were collected from the antecubital vein between 8 to 10 a.m., in a sitting position after 12 hours of fasting and avoiding of ethanol. For the determination of plasma fibrinogen blood was anticoagulated with 3.8% trisodium citrate (9:1 vol/vol) and cooled on ice until centrifugation. For determination of homocysteine, blood was collected in a cool vacutainer containing EDTA, which was stored on ice for a maximum of 2 hours till the centrifugation at 3000 g for 5 minutes at 4°C. Plasma homocysteine levels were measured with an automatic Abott Axsym analyzer, which is based on the technology of polarized immunofluorescence. The intra and inter-assay coefficients of variation of homocysteine did not exceed 5%.

Arterial blood pressure was automatically measured at the end of the physical examination with subject in sitting position. Hypertension was defined as a systolic blood pressure >/= 140 mmHg, a diastolic blood pressure >/= 90 mmHg, or the use of any antihypertensive medication; hypercholesterolemia was defined as total cholesterol levels greater than 220 mg/dl or the use of lipid lowering agents and diabetes mellitus as a fasting blood glucose > 125 mg/dl or the use of antidiabetic medication.

### Statistical analysis

Continuous variables are presented as mean values ± standard deviation, while qualitative variables are presented as absolute and relative frequencies. Associations between categorical variables were tested by the use of contingency tables and the calculation of chi-squared test. Comparisons between normally distributed continuous variables and categorical variables were performed by the calculation of Student's t-test and multi way Analysis of co-Variance (multi-ANCOVA), after controlling for homoscedacity and various potential confounders. In the case of asymmetric continuous variables the tested hypotheses were based on the calculations of non-parametric tests, such as Mann – Whitney and Kruskal – Wallis. Kolmogorov-Smirnov criterion assessed normality of continuous variables. Finally, correlations between continuous variables were tested through multiple regression analysis after the adjustment for the potential confounders and interactions. The J- shape association between the exposure variable (ethanol intake) and homocysteine levels was illustrated by connecting the mean values of the investigated parameters using 3^rd ^order interpolating polynomials.

All reported *P*-values are based on two-sided tests and compared to a significance level of 5%. However, due to multiple significance tests we used the Bonferroni correction (since the number of comparisons was less than ten) in order to account for the increase in Type I error. SPSS 11.0 software (SPSS Inc. 2002, Illinois, USA) was used for all the statistical calculations.

## Results

Thirty four percent of males and 62% percent of females reported ethanol abstinence within the recorded 7-day period (p < 0.001). In addition, 37% of males and 33% of females consumed < 12 gr of ethanol per day, 16% of males and 4% of females consumed 12 – 24 gr of ethanol per day, and 13% of males and 1% of females consumed > 24 gr of ethanol per day (1.6% of males and 0.4% of females consumed > 48 gr/d), during the preceding week. Furthermore, middle-aged male participants (45 – 65 years old) consumed higher quantities of ethanol compared to younger (< 45 years) or older individuals (18 ± 16 vs. 12 ± 14 vs. 15 ± 16 gr of ethanol per day, respectively, p = 0.002), while no statistically significant differences were observed between ethanol consumption and age, in females (9 ± 13 vs. 11 ± 12 vs. 12 ± 14 gr of ethanol per day, respectively, p = 0.391). Ethanol intake comes from wine in 65% of men and 77% of women, from beer in 22% of men and 11% of women and from spirits or other drinks 13% of men and 12% of women. Further, descriptive characteristics of the studied population by ethanol consumption level are presented in Table 1. By the exception of years of school (p = 0.02) and prevalence of hypertension (p = 0.01) no other associations were observed between ethanol intake and smoking habits, prevalence of hypercholesterolemia, diabetes and obesity.

**Table 1:** Descriptive characteristics of study's participants by alcohol intake, and by gender

	Daily ethanol intake				
Males	None	< 12 gr/d	12 – 24 gr/d	25 – 48 gr/d	> 48 gr/d
Current smoking	39%	54%	43%	49%	54%
Physical inactivity	62%	56%	67%	69%	64%
Years of school (SD)	14(4)	13(4)	11(6)	11(4)**	9(4)**
Hypertension	28%	37%**	39%**	45%**	44%**
Hypercholesterolemia	33%	34%	44%	39%	34%
Diabetes	11%	11%	9%	9%	7%
Obesity	22%	24%	23%	19%	24%
Females					
Current smoking	38%	25%	32%	37%	30%
Physical inactivity	64%	75%	62%	57%	80%
Years of school (SD)	13(4)	12(4)	11(3)*	10(4)**	8(3)**
Hypertension	17%	28%**	22%	22%	26%**
Hypercholesterolemia	28%	35%	32%	37%	40%
Diabetes	8%	10%	12%	6%	6%
Obesity	18%	15%	12%	17%	20%

** Bonferroni α < 0.01 and * α < 0.05 for the comparisons between ethanol intake and no intake groups.

Homocysteine values were higher in males as compared to females (14.5 ± 6 vs. 10.8 ± 3.5 μmol/L, p < 0.001). The 10^th ^percentile for men was 8.6 μmol/L and for women 6.8 μmol/L, while the 90^th ^percentiles were 18 μmol/L and 14 μmol/L, for men and women, respectively. Due to the significant differences observed between genders in homocysteine levels, all the following analyses will be gender-specific.

**Figure 1:**
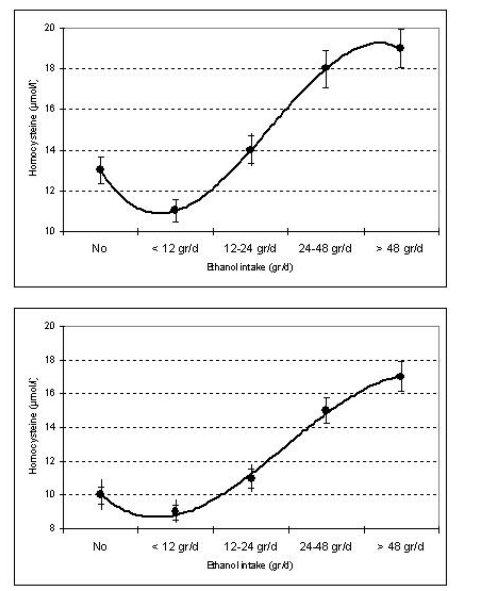
Homocysteine levels by daily ethanol intake in males (upper figure) and females (power figure) (continuous line is a 3^rd ^order interpolating polynomial)

Unadjusted analysis revealed a J-shape association between ethanol quantities consumed during the past week (none, < 12 gr, 12 – 24 gr, 25 – 48 gr, >48 gr of ethanol per day) and homocysteine levels in both males (13 ± 3 vs. 11 ± 3 vs. 14 ± 4 vs. 18 ± 5 vs. 19 ± 3 μmol/L, respectively, p < 0.01) and females (10 ± 4 vs. 9 ± 3 vs. 11 ± 3 vs. 15 ± 4 vs. 17 ± 3 μmol/L, respectively, p < 0.01). Post hoc analysis revealed that the lowest values of homocysteine levels were observed in people who reported moderate daily ethanol intake of <12 gr (Bonferonni α = 0.02 for males and α = 0.02 for females). No differences were observed when we stratified our analysis by alcoholic beverages primarily consumed. Figure 1 illustrates the observed J-shape association between ethanol intake and homocysteine levels in males and females.

However, since several potential confounders may influence the relationship between ethanol intake and homocysteine concentration we repeated our analysis after taking into account age, gender, pack-years of smoking, presence of hypertension, hypercholesterolemia, and diabetes, body mass index, fruits and vegetables consumption, especially leafy green vegetables, legumes, citrus fruits and juices that are reached in folic acid, as well as years of school. Multivariate regression analysis showed that <12 gr/d ethanol intake was inversely associated with homocysteine levels (b-coefficient = -0.5, p = 0.02) as compared to no consumption. On the other hand, increased ethanol intakes, i.e. 12 – 24 gr/d, 24 – 48 gr/d or > 48 gr/d were positively associated with homocysteine concentration (b-coefficient = 1.2, p = 0.03, b-coefficient = 1.8, p = 0.02 and b-coefficient = 1.9, p = 0.02, respectively). No differences were observed when we stratified our analysis by gender.

## Discussion

The results of the present study revealed a J-shape association between ethanol consumption and homocysteine levels, of a large, random and population representative sample, free of cardiovascular disease. The lowest values of homocysteine were observed in daily ethanol intake of less than 12 gr, both in men and women and remained significant after adjustment for several potential confounders.

Our results are in line with that of some other studies. For example, De Bree et al. [16] observed lower homocysteine concentrations at higher levels of ethanol consumption, with non drinkers having a (geometric) mean homocysteine of 14.2 μmol/L, compared to 13.9 μmol/L in drinkers of ≤ 20 gr ethanol/ day, 12.5 μmol/L in drinkers of between 20 and 40 gr/day and 13.1 μmol/L in drinkers of ≥ 40 gr / day. In our study, the lowest homocysteine concentrations were observed with ethanol intakes <12 gr /day. This difference between our and the previous study may attribute to the type of alcoholic beverage consumed, since in the study of Bree et al. beer was the main alcoholic drink, while in our study it was wine. In another study the most positive association of ethanol (from beer consumption) on homocysteine levels was observed at ethanol intakes 4 to 14 gr/d [17]. Another study in severely obese patients revealed a U-shaped association between homocysteine concentrations and the amount of ethanol consumption [18]. In particular, the most beneficial effect was observed with consumption of < 100 gr ethanol/ week and especially in red wine consumers, compared to subjects who consumed white wine, beer or spirits. However, the lower homocysteine concentrations in those consuming less than 100 gr ethanol/ week were not significant after controlling for serum folate concentration. Finally a study in elderly subjects also found a J-shape relation, with nondrinkers and subjects consuming ≥ 60 drinks/ month, showing higher homocysteine concentrations, compared to those consuming ≤ 60 drinks/ month [19]. However, the interpretation of the results from the previous study is difficult because the total amount of ethanol ingested was not calculated.

On the contrary, there are several studies that have shown a linear relationship between ethanol intake and homocysteine levels. For example, Folsom et al. [20] in a study of middle-aged men and women showed a positive association of ethanol on homocysteine. However, he studied very low intakes of ethanol, ranging from 27 to 47 gr/ week, and this may be the reason why a J -shaped association was not observed. According to our findings, a significant positive association was observed at much higher intakes (i.e. 84–168 gr/week). Another study in young women (aged 15–44) [21] showed that those consuming >7 drinks/ week were 90% more likely to have elevated homocysteine levels (> 10 μmol/l), compared to those who did not consume ethanol. In the same study, subjects consuming 1–7 drinks/week had the same homocysteine levels with those that didn't consume, supporting, partially, two relations between ethanol intake and homocysteine. However, the association between ethanol and homocysteine levels failed to achieve statistical significance. Finally, homocysteine was positively associated with ethanol intake in the Framingham Offspring cohort [22] at daily intakes of more than 15 g. In this study liquor and red wine consumption was significantly and positively associated with homocysteine. This association was not observed with beer and white wine consumption.

Our data were analyzed according to total ethanol intake and did not distinguish between different types of ethanol. Rimm et al. [23]reviewed the literature with respect to beverage-specific effects on coronary heart disease and could not find any systematic effects. On the contrary, they showed that the U-shaped relation between ethanol intake and cardiovascular disease mortality persisted in populations with very different drinking patterns. Although there have been many publications on this topic since the aforementioned review, no systematic pattern or results have emerged until now. Perhaps most notably in this respect are the findings which suggest similar protective effects of ethanol not only in Bavaria (Germany) and the Czech Republic, where beer is mainly consumed, but also in Mediterranean countries, where wine is the most popular alcoholic beverage [24]. Additionally, Greece is a Mediterranean country, where wine is the most commonly used alcoholic beverage. According to our findings as well as the recent results from the EPIC-Greece study [25] 72% of women's total ethanol intake comes from wine, 26% from beer and 12% from spirits. For men wine contributes to 56% of total ethanol intake, beer 15% and aniseed drinks 20%. Therefore our data do not support the assumption of Mennen et al. [26] who suggested that the inverse association between ethanol and homocysteine is seen in populations which consume predominantly beer.

Chronic alcoholism has been found to be associated with hyper-homocysteinaemia, which could attribute to disturbed folate metabolism and to changes in circulating concentrations of vitamin B_12 _and pyridoxal phosphate, as well as to ethanol intake per se [27]. Finally, the dual effect of ethanol consumption on homocysteine has also been confirmed from data of animal studies, which clearly show effects of excessive ethanol intake on the methionine cycle [13]. Nevertheless, the finding that subjects who do not consume ethanol have higher homocysteine levels than light to moderate drinkers needs further investigation. Whether this fact can be attributed to ethanol per se or to other substances of alcoholic beverages (e.g. folate, B_12_, B_6_, betaine) remains unclear and more intervention and experimental studies are necessary.

## Limitations

This study as a cross-sectional one cannot establish causal relations but only generate hypothesis for associations. The population studied in this work is homogeneous and may reflect lifestyle habits in similar cultures, like Western Europe, Mediterranean etc. However, our findings could not extrapolate into other populations without further investigation and consideration. Also, the numbers of participants in categories of high intake (>48 gr of ethanol /d) were rather small, and the impression of the effects in homocysteine levels in even higher ethanol consumption may be misleading. Although this analysis has been adjusted for several known confounders, we have indirectly investigated the impact of serum folate and vitamins B_6 _and B_12 _intake (through food groups consumed) on homocysteine concentrations. In addition, kidney function is a strong determinant of homocysteine; however, we have not measured serum creatinine. The later may be another limitation of our study. Additionally, misreporting of ethanol consumption, due to social class can be a potential confounder.

## Conclusion

The present study supports the existence of a J-shape association between ethanol consumption and homocysteine levels in both males and females, of a large, random and population representative sample, free of cardiovascular disease. Therefore our results indicate that daily consumption of 1–2 units of ethanol is associated with lower homocysteine concentrations and provide further evidence for a variant association between ethanol intake and coronary heart disease risk in both genders.
